# Self-Assembly of Amphiphilic Comb-like Copolymers into Micelles and Vesicles in Solution

**DOI:** 10.3390/polym17131870

**Published:** 2025-07-04

**Authors:** Qiaoyue Chen, Kun Tian, Ruiqi Zhu, Mingming Ding, Zhanwen Xu

**Affiliations:** 1 Xinjiang Laboratory of Phase Transitions and Microstructures in Condensed Matters, College of Physical Science and Technology, Yili Normal University, Yining 835000, China; chenqy210701@163.com (Q.C.); rqzhu135@163.com (R.Z.); 2 State Key Laboratory of Molecular Engineering of Polymers, Key Laboratory of Computational Physical Sciences, Department of Macromolecular Science, Fudan University, Shanghai 200433, China; 23210440020@m.fudan.edu.cn; 3 School of Chemical Engineering and Light Industry, Guangdong University of Technology, Guangzhou 510006, China

**Keywords:** amphiphilic comb-like copolymers, micelles, vesicles, Brownian dynamics

## Abstract

Combining Brownian dynamics simulations and self-consistent field theory, we demonstrate that stable assembled structures, such as vesicles, toroidal micelles, bowl-like micelles, sheet-like micelles, non-spherical vesicles, and cylindrical micelles, are dependent on the molecular parameters of amphiphilic comb-like copolymers. Importantly, we find that vesicle formation involves two intermediate states, sheet-like and bowl-like micelles, and the difference in their free energies is minimal, which illustrates the coexisting phase between them. Moreover, the assembled vesicles can be modulated in the membrane thickness with overall size, unchanged only by adjusting the backbone length. We also demonstrate the coexistence of toroidal and cylindrical micelles because neither structure has a significant advantage over the other in free energy. Our work points out how to obtain different morphologies by adjusting the molecular parameters of amphiphilic comb-like copolymers, instilling confidence in their potential for stable drug encapsulation and enhanced targeted drug delivery.

## 1. Introduction

Copolymer self-assembly has been a topic of great interest because of its ability to form functional micelles or vesicles, which, in turn, determine the potential applications of copolymers [1,2,3,4,5]. For example, spherical micelles and vesicles exhibit stable drug encapsulation and enhanced targeted drug delivery [6,7,8,9,10]. Toroidal micelles have applications in delivery systems that mimic bacteriophages and vertebrate DNA rings [11,12]. In biomedical sensing, self-assembled length-controlled cylindrical micelle brushes are used to enhance the signal recognition capability of detectors [13].

The self-assembled morphologies of copolymers in solution are influenced by numerous factors, with molecular structure being the most intrinsic. To date, the self-assembling mechanisms of linear block copolymers [7,14,15], comb-like copolymers [16,17,18,19], hyperbranched copolymers [20,21], and other topologically structured copolymers continue to be subjects of ongoing exploration in both simulation and experimental studies [22]. Among them, the self-assembly of non-linear polymers, such as graft copolymers, emerges as a more promising component in self-assembled materials [23,24,25,26,27,28,29,30]. This is attributed to their lower critical micelle concentration [31] and higher interfacial stability [32], making them withstand rigorous environments such as high pressure and heat, and strong alkalis and acids [33,34].

Given this, many researchers have used different graft molecular models to explore their self-assembly morphologies. For instance, Wang et al. employed dissipative particle dynamics (DPD) simulations to investigate the self-assembly of amphiphilic comb-like copolymers, specifically using the $PA_{6}{\left(B_{2}\right)}_{3}$ and $QC_{6}{\left(D_{2}\right)}_{3}$ models (the subscript numbers 6, 2, and 3, respectively, represent the backbone length, side-chain length, and grafting number), which results in monolayer vesicles [35]. Lin et al. combined self-consistent field theory (SCFT) with DPD simulations to study the $A_{9}$-graft-${\left(B_{6}\right)}_{3}$ model, revealing the formation of segmented, multi-layered composite vesicles [10]. Liu et al. focused on the spontaneous formation and fusion of onion-like vesicles using DPD simulations with the $A_{6}{\left(B_{2}\right)}_{3}$ model [19]. Additionally, Khokhlov et al. used Monte Carlo (MC) simulations to examine the conformational transitions of a single amphiphilic comb-like copolymer, observing structural changes from spherical to elongated ellipsoids and pearl-necklace configurations as the grafting density increased [36]. Sheng and Tsao et al. separately employed comb-like graft copolymers composed of a backbone of poly((N-vinylcarbazole)-co-(4-vinylbenzyl chloride)) (P(NVK-co-VBC)) and side chains of poly(((2-dimethylamino)ethyl methacrylate)-co-(tert-butyl acrylate)) (P(DMAEMA-co-tBA)) to regulate the formation of vesicles from unilamellar to multilamellar structures using the DPD method. They found that variations in grafting density can modulate the thickness and the number of layers in the hydrophobic region [37,38]. These studies highlight the critical role of molecular model selection in determining the self-assembly morphologies of amphiphilic comb-like copolymers. However, a significant challenge remains: the diversity of molecular models makes direct comparisons between different studies difficult. This limitation hinders the ability to establish a precise relationship between molecular parameters and the resulting self-assembled morphologies.

In the current study, we systematically investigate the relationship between the molecular parameters and self-assembled morphologies of amphiphilic comb-like chains in solution by employing Brownian dynamics (BD) simulations. The objective is to control the self-assembly structures by tuning these molecular parameters for diverse applications. The study revealed that various self-assembled morphologies could be observed, including toroid micelles, bowl-like micelles, vesicles, sheet-like micelles, cylindrical micelles, and non-spherical vesicles, by adjusting the length of the backbone and side chains, and the grafting density. In addition, we reveal the transition mechanisms between different morphologies by calculating the free energy of different self-assembled morphologies using SCFT. The remainder of this article is organized as follows. Section 2 provides the model and simulation details. Section 3 constructs the morphological phase diagram, elucidates the self-assembly mechanism, and provides the relationship between the molecular parameters and self-assembled morphologies. Section 4 presents the conclusion and future outlook.

## 2. Model and Methods

**Figure 1:**
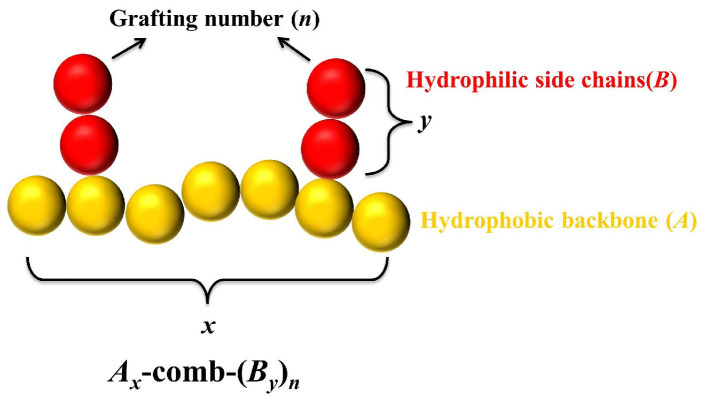
Coarse-grained model of amphiphilic comb-like copolymers: $A_{x}$-comb-${\left(B_{y}\right)}_{n}$, composed of a hydrophobic backbone with a length of *x* (represented by *A*, yellow) and *n* hydrophilic side chains with a length of *y* (represented by *B*, red), where n≤x.

**BD simulations:** We conduct BD simulations to investigate the self-assembly dynamics of amphiphilic comb-like chains. As demonstrated in our previous work and related simulations, the coarse-grained model is employed, which can reasonably capture the essential interactions and structural features with the reduced unit system [31,32,39,40,41,42,43,44,45]. As illustrated in Figure 1, the amphiphilic comb-like chains are modeled as bead-spring chains: The hydrophilic side chains *B* (red) are randomly grafted onto the hydrophobic backbone *A* (yellow). We denote amphiphilic comb-like chains as $A_{x}$-comb-${\left(B_{y}\right)}_{n}$, where *x* and *y* represent the lengths of the backbone and side chains, respectively, and *n* denotes the grafting number. All bonded interactions, including the adjacent beads of the backbone and the side chains, and the grafting points between the beads of the backbone and the side chains, are calculated by the finite extensible nonlinear elastic (FENE) potential [46], i.e.,

(1) $$U_{FENE}\left(r\right)=-0.5\kappa R_{0}^{2}\ln\left[1-{\left(r/R_{0}\right)}^{2}\right]$$

where κ denotes the spring constant and $R_{0}$ is the maximum separation distance for the bead–bead distance *r*.

The non-bonded interactions between two beads accounted for the excluded-volume interactions described through the Lennard-Jones (LJ) potential, i.e.,

(2) $$U_{LJ}\left(r\right)=4\epsilon \left[{\left(\sigma /r\right)}^{12}-{\left(\sigma /r\right)}^{6}\right]$$

Here, ϵ and σ represent the energy and length parameters, respectively. Implicit solvents are used in the simulation system. For the interactions between hydrophobic beads, we specify a cutoff distance $r_{c}=2.5\sigma$ for the LJ potential, corresponding to a poor solvent condition [14,47]. For the other pairwise beads, we set the cutoff to $r_{c}=2^{1/6}\sigma$, corresponding to a good solvent condition [48]. Additionally, both LJ potentials are shifted separately to ensure $U_{\mathrm{LJ}}=0$ at $r_{c}$.

We employ BD simulations, where the motion for bead *i* is given by [49]

(3) $${\dot{r}}_{i}\left(t\right)=-\frac{1}{\xi }\nabla U_{i}+\frac{1}{\xi }f_{i}\left(t\right)$$

$U_{i}$ represents the potentials from all FENE and LJ interactions as discussed, and ξ denotes the friction coefficient experienced by a bead in the solvent. The random force $f_{i}\left(t\right)$ is generated based on the thermal bath model using a Gaussian-distributed random number generator, satisfying the fluctuation–dissipation theorem: $\langle f_{i}\left(t\right)f_{j}\left(t^{\prime }\right)\rangle =2\xi k_{B}T\delta_{ij}\delta \left(t-t^{\prime }\right)I$, where $k_{B}$ is Boltzmann’s constant, *T* is the temperature, and I denotes the unit tensor. Equation (3) neglects inertial effects and can be efficiently integrated using the Euler method. In this work, we disregard hydrodynamic interactions between different beads, which enables us to compute the steps for a longer time with reduced computational effort.

We adopt length, mass, and energy units as σ, *m*, and ϵ, respectively. Consequently, the unit of time can be described as $\tau ={\left(m\sigma^{2}/\epsilon \right)}^{1/2}$. We set the temperature as $k_{B}T=1.0$, the time step as $\Delta t=10^{-4}$, and the friction coefficient as ξ=0.5. Furthermore, we fix the parameters of the FENE potential as κ=30 and $R_{0}=1.5$ to prevent chain crossing [50]. We conduct simulations within a 20σ×20σ×20σ cubic box with periodic boundary conditions. All samples are fixed with the same volume fraction 0.15 and are simulated with 3.5–$5.0\times 10^{8}$ time steps. In addition, independent simulations are carried out 5–10 times with different random seeds.

**SCFT:** In the SCFT calculations, we consider an incompressible blending system composed of AB comb-like copolymers and solvent molecules of short A homopolymer. Under the approximations of the mean-field treatment and Gaussian-chain model, the free energy in the canonical ensemble can be expressed as

(4) $$\begin{array}{cc} \; & \frac{NF}{\rho_{0}Vk_{B}T}=-\phi \ln\frac{Q_{C}}{\phi }-\frac{1-\phi }{\gamma }\ln\frac{Q_{H}}{1-\phi }\\ \; & +\;\frac{1}{V}\int dr\{\chi N\phi_{A}\left(r\right)\phi_{B}\left(r\right)-\omega_{A}\left(r\right)\phi_{A}\left(r\right)-\omega_{B}\left(r\right)\phi_{B}\left(r\right)\\ \; & -\;\eta \left(r\right)[1-\phi_{A}\left(r\right)-\phi_{B}\left(r\right)]\} \end{array}$$

where $\omega_{K}\left(r\right)$ is the mean-field conjugating to the volume fraction $\phi_{K}\left(r\right)$ (K=A or B). The spatial function η(r) is a Lagrange multiplier used to enforce the incompressibility condition, $\phi_{A}\left(r\right)+\phi_{B}\left(r\right)=1$. The two quantities $Q_{C}$ and $Q_{H}$ are the partition functions of single copolymer chain and single homopolymer chain interacting with the mean fields of $\omega_{A}\left(r\right)$ and $\omega_{B}\left(r\right)$, respectively.

The A_1_-comb-(B_1_)_1_, which can be seen as the repeating units of A_*x*_-comb-(B_*y*_)_*n*_ at y=n=1 in the BD simulation, is modelled as an AB_2_ star copolymer with $f_{A}=0.5$ and *N* segments in the SCFT. In other words, 0.5N segments of the comb-like copolymer in the SCFT correspond to 1 bead of the copolymer model in the BD. By setting the number of total segments for the solvent to be 0.2N and the interaction parameter between A and B components $\chi_{\mathrm{AB}}N$ to be 15 ($\chi_{\mathrm{AB}}$ denotes the Flory–Huggins parameter), we observed the order–disorder transitions and self-assembled structures in the SCFT similar to that in the BD. More details of the SCFT can be found in references [51,52] or the Supporting Information.

## 3. Results and Discussion

**Figure 2:**
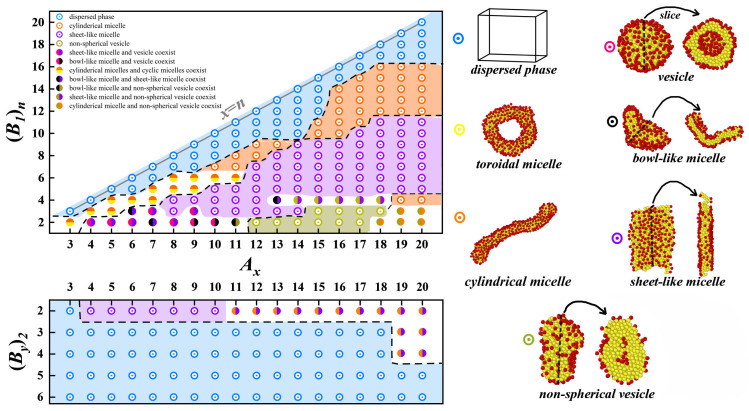
Phase diagrams of $A_{x}$-comb-${\left(B_{1}\right)}_{n}$ and $A_{x}$-comb-${\left(B_{y}\right)}_{2}$, respectively, where the blue region represents the dispersed phase, the yellow region denotes toroidal micelle, the orange region indicates cylindrical micelle, the pink region represents vesicle, the black region signifies bowl-like micelle, the purple region denotes sheet-like micelle, and the gray region indicates non-spherical vesicle. The corresponding simulation snapshots for each region are indicated on the right, where the arrows point to the cross-sectional slice of the structures. The two colored circles represent the coexistence state of two structures simulated under the same starting conditions.

As shown in Figure 2, we initially fix y=1, that is $A_{x}$-comb-${\left(B_{1}\right)}_{n}$, to explore the possible self-assembling structures. The results reveal that when n=x, the system cannot form a stable structure and is in the dispersed phase. At this time, each bead on the backbone is grafted with a side chain. As we reported earlier, at this time, the hydrophobic backbone chain is surrounded by the hydrophilic side chains to form a better encapsulation layer, that is to say, the formation of a single molecule micelle [40], which leads to the difficulty of further self-assembly with other chains. As *n* decreases, we begin to be able to observe various structures. For example, first, one can self-assemble to form cylindrical micelles; however, when *x* is small, the coexistence of cylindrical micelles and toroidal micelles can also be observed. The next larger percentage is sheet-like micelles. In addition, when n<4, the structures obtained are more abundant; for example, the coexistence of sheet-like micelles and vesicles, and non-spherical vesicles and cylindrical micelles can be obtained when *x* is small.

We then fix n=2, that is, $A_{x}$-comb-${\left(B_{2}\right)}_{2}$, to investigate the self-assembling structures with two side chains. Our results indicate that most stable self-assembled structures cannot be formed. This implies that the desired self-assembled structure will be formed only if the molecular parameters are tuned to a suitable range. We find that only sheet-like micelles and cylindrical micelles can be formed with small *y*. When *x* is larger, it is in the coexistence phase of sheet-like micelles and cylindrical micelles. As reported in the literature, stabilized micelles and vesicles with different shapes have very many promising applications [6,7,8,9,10]. Therefore, we proceed to further analyze the self-assembly process of toroidal micelles, cylindrical micelles, and vesicles, as well as the free energy of their structures.

**Figure 3:**
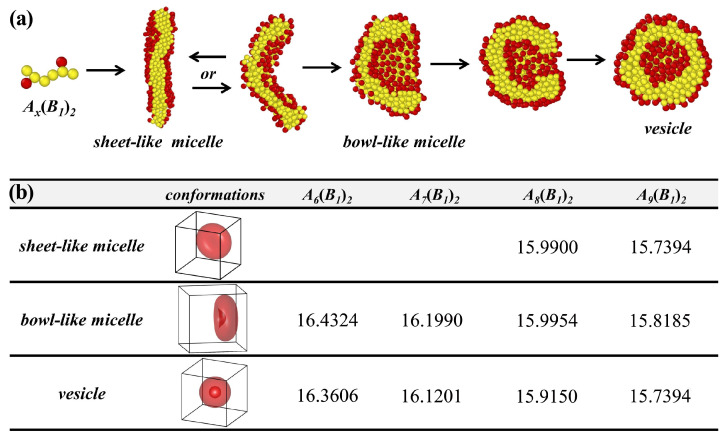
(**a**) Self-assembly roadmap of amphiphilic comb-like copolymers with the molecular parameters $A_{x}{\left(B_{1}\right)}_{2}$, which indicates that they first form sheet-like micelles, then the structure bends and forms bowl-like micelles, or may revert to sheet-like micelles, and finally the structure closes to form vesicles. (**b**) Free energies calculated by SCFT for the sheet-like micelles, the bowl-like micelles, and the vesicles correspond representatively to (**a**).

We trace the dynamic process of vesicle formation, as shown in Figure 3a. The chains rapidly aggregate into numerous small clusters at the initial stage and assemble into sheet-like micelles. At this stage, two distinct scenarios emerge, one where the system always maintains the sheet-like structure, accompanying occasional minor bending. The other is the gradual bending of the sheet-like micelles, transitioning into bowl-like structures. Similarly, the system either remains in the bowl-like micelles or the bowl’s opening gradually narrows until completely closed, forming a spherical vesicle. This suggests that vesicle formation involves two intermediate states, sheet-like and bowl-like micelles, rather than direct formation. This closely resembles the findings of Gianneschi et al., who utilized in situ liquid-cell transmission electron microscopy to observe the formation of bicontinuous micelle structures during the transition of amphiphilic block copolymer micelles into higher-order vesicle structures [53].

As shown in Figure 2, the simulation results indicate the coexistence of vesicles, bowl-like micelles, and sheet-like micelles. To justify this coexistence of states, we calculate their formation and the corresponding free energies using SCFT, as shown in Figure 3b. Consistently, we observe the coexistence of vesicles and bowl-like micelles as well as sheet-like micelles, except that $A_{6}{\left(B_{1}\right)}_{2}$ and $A_{7}{\left(B_{1}\right)}_{2}$ failure to form the sheet-like micelles. Although the results of the coexisting phases do not fully agree with the simulation results of BD, the results show that the difference in the free energy of the different phases is minimal for the same molecular parameters, which confirms that this coexisting phase is reasonable. It also shows that this system’s potential barrier for vesicle formation is low, which opens up new possibilities for self-assembly to form vesicles.

**Figure 4:**
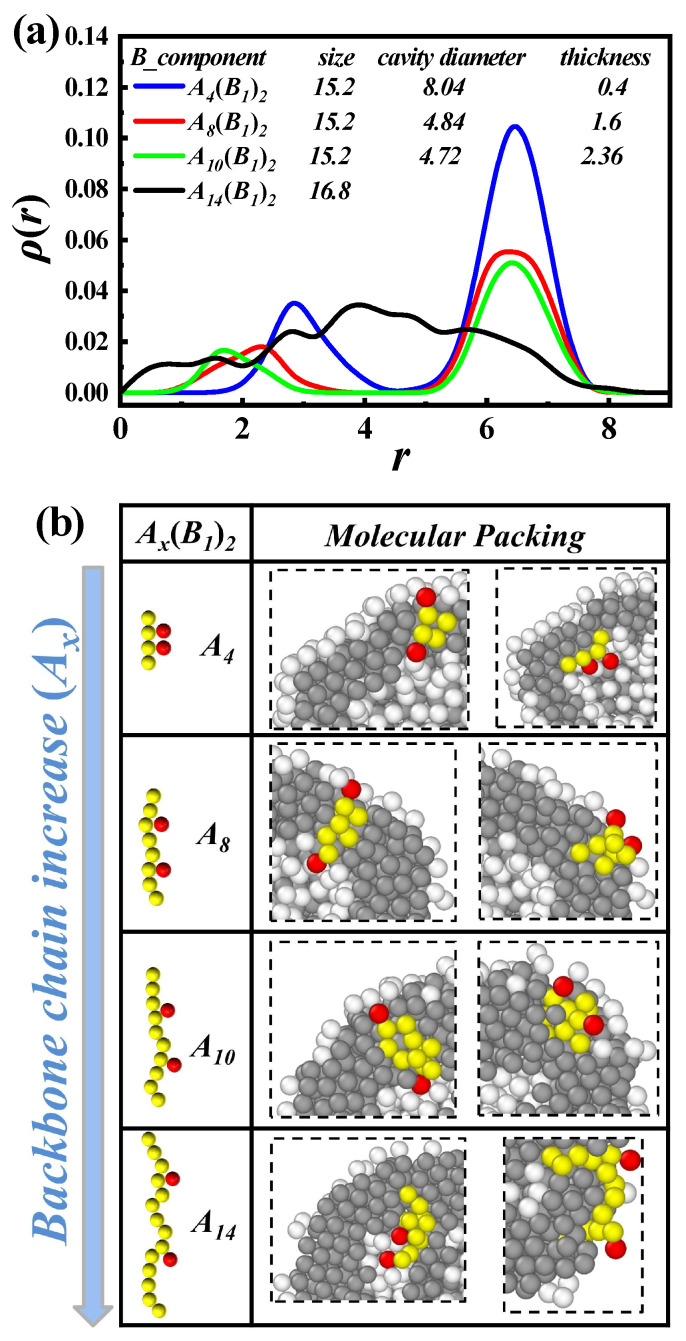
(**a**) Average density [ρ(r)] of the hydrophilic beads (B) as a function of the distance *r* from the center of the assembled vesicles, which are composed of $A_{x}{\left(B_{1}\right)}_{2}$ and x=4, 8, 10, and 14, respectively. The overall size of the vesicles, the cavity diameters, and the membrane thicknesses are also listed. (**b**) Amplified molecular packing of the vesicles corresponds to the cases of (**a**).

The control of vesicle size and membrane thickness has been a widely studied topic [8,10,35,54,55]. In Figure 4a, we statistically analyze the density distribution of self-assembled vesicles with molecular parameters of $A_{x}$-comb-${\left(B_{1}\right)}_{2}$, where x=4, 8, 10, and 14, respectively. Here, *r* represents the distance from the vesicle center, and ρ(r) denotes the average density of the vesicle at r+Δr on the spherical shell. Specifically, the vesicle’s center of mass is first determined. Then, the number of hydrophilic beads at different radial distances within concentric spherical shells of equal thickness is counted to obtain the distribution profile. We observe two peaks with increasing *r*, except $A_{1}4{\left(B_{1}\right)}_{2}$, which further demonstrates the formation of vesicles. When x=14, corresponding to non-spherical vesicles, the density distribution no longer follows the pattern of two peaks. As *x* increases, the first peak continuously shifts towards the vesicle’s center. However, the second peak is almost unchanged, suggesting that the membrane thickness is increasing while the overall dimensions remain constant, which matches the calculated values. This is a promising method for controlling vesicle size to modulate membrane thickness, which infers that the assembled vesicles formed by comb-like copolymers with larger gap lengths (i.e., fewer grafting points) possess thicker hydrophobic membranes and smaller cavities.

To clarify the molecular mechanism of such size-invariant but membrane-thickness-variable vesicles, we give amplified molecular packing, as illustrated in Figure 4b. When x=4, due to the high graft density of hydrophilic side chains, most hydrophobic backbones exhibit a straighter shape, and the average distance from backbone end to end is approximately 4. However, when x=10, with side chains spaced farther apart, the backbone tends to bend, and the average distance from the backbone end to end is approximately 2.36. As hydrophobic backbones tend to aggregate, thicker hydrophobic membranes are formed. Furthermore, when increased to “overloaded” lengths, such as x=14, the hydrophobic portion of the packing is not constrained, resulting in a non-spherical vesicle.

**Figure 5:**
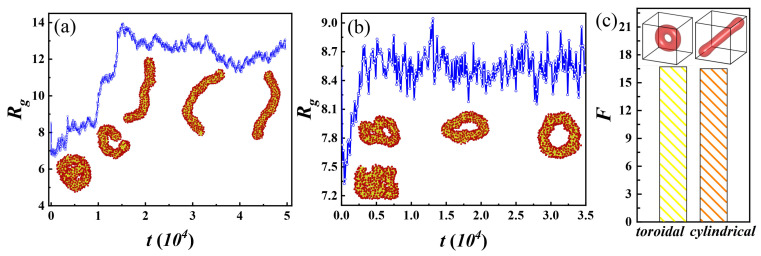
Gyration radius ($R_{g}$) during the formation of cylindrical micelles (**a**) and toroidal micelles (**b**) as a function of simulation time (*t*). The insets show representative simulation snapshots captured during the self-assembly process. (**c**) Free energy (*F*) for toroidal and cylindrical micelles, where the insets exhibit stable states.

Figure 2 shows a large coexistence zone for toroidal and cylindrical micelles. To explore their formation mechanism and to verify the rationality of their coexistence, we calculate the radius of gyration during their formation respectively and calculate the free energy of their steady states using SCFT, as shown in Figure 5. The comb-like chains rapidly aggregate at the initial simulation. Partial aggregates gradually become straight, which may be attributed to the rearrangement of comb-like chains, causing the formation of cylindrical micelles. Differing from that of cylindrical micelles, the middle of the aggregate gradually broke open, eventually forming toroidal micelles. As depicted in Figure 5a,b, when the structure of the toroidal and cylindrical micelles is gradually stabilized, their radii of gyration are also only stable, accompanied by small fluctuations. We then calculate their free energies using SCFT in Figure 5c and find their values almost identical. This suggests that under the same molecular parameter conditions, neither structure has a significant advantage over the other, and thus, coexisting phases can easily occur.

## 4. Conclusions

In this work, we systematically investigate the relationship between the molecular parameters and self-assembled morphologies of amphiphilic comb-like chains $A_{x}$-comb-${\left(B_{y}\right)}_{n}$ in solution by employing BD and SCFT. The results indicate that the adjustments in *x* and *n* lead to different arrangements of chains, resulting in stable states such as vesicles, toroidal micelles, bowl-like micelles, sheet-like micelles, non-spherical vesicles, and cylindrical micelles. Importantly, we find that vesicle formation involves two intermediate states, sheet-like and bowl-like micelles, and the difference in their free energies is minimal, which illustrates the coexisting phase between them. Moreover, the assembled vesicles can be modulated in the membrane thickness with overall size unchanged by only adjusting the backbone length. We also demonstrate the coexistence of toroidal and cylindrical micelles because neither structure has a significant advantage over the other in free energy. Our work shows how to obtain different morphologies by adjusting $A_{x}$-comb-${\left(B_{y}\right)}_{n}$, especially the size-invariant but membrane-thickness-variable vesicles and toroidal and cylindrical micelles, which provides insight into their applications such as stable drug encapsulation and enhanced targeted drug delivery. Of course, the interaction strength of hydrophobic beads is another critical factor influencing the outcomes, alongside molecular parameters. The synergistic effects of these factors warrant further investigation. Additionally, the availability of efficient sampling methods to obtain the distribution of coexisting phases is a question worth exploring. Furthermore, future studies could extend to environmentally responsive dynamic assembly, investigating phenomena such as temperature-gradient-induced dynamic phase transitions.

## Data Availability

The original contributions presented in this study are included in the article/Supplementary Material. Further inquiries can be directed to the corresponding author.

## Supplementary Materials

The following supporting information can be downloaded at: https://www.mdpi.com/article/10.3390/polym17131870/s1, SCFT Method.
