# Supplementary material for: Self-Assembly of Amphiphilic Comb-like Copolymers into Micelles and Vesicles in Solution
Source: Polymers (Basel). 2025 Jul 4;17(13):1870. doi: 10.3390/polym17131870 (PMC12251614; doi:10.3390/polym17131870)
Supplement: Supplementary file 1 [file polymers-17-01870-s001.zip › polymers-3672694-supplementary.pdf]

# Supporting Information: Self-assembly of amphiphilic comb-like copolymers into micelles and vesicles in solution

Ruiqi Zhu,[†,§] Kun Tian,[‡,§] Mingming Ding,[*,¶,†] and Zhanwen Xu[*,‡]

*†Xinjiang Laboratory of Phase Transitions and Microstructures in Condensed Matter Physics, College of Physical Science and Technology, Yili Normal University, Yining 835000, China*

*‡State Key Laboratory of Molecular Engineering of Polymers, Key Laboratory of Computational Physical Sciences, Department of Macromolecular Science, Fudan University, Shanghai 200433, China*

*¶School of Chemical Engineering and Light Industry, Guangdong University of Technology, Guangzhou 510006, China*

*§R. Zhu and K. Tian contributed equally to this work*

E-mail: mmding@gdut.edu.cn; xuzhanwen@fudan.edu.cn

This PDF file includes:

SCFT Method

## SCFT Method

In the SCFT calculations, we consider a system with volume of $V$ consisting of $n_1$ comb-like copolymers and $n_2$ solvent molecules of short A-homopolymers, respectively. The total number of segments of comb-like copolymers is specified as $N$ which having $N_A = fN$ A-segments, and that of A-homopolymer is denoted by $N_H = \gamma N$. The total number of the graft of the comb-like copolymer is $m$, and each graft consists of $N_B$ B-segments, and then $N = N_A + mA + mN_B$. Simply, we assume that all segments have equal length $b$ and density $\rho_0$, thus having $n_1 N + n_2 N_{\mathrm{H}} = V\rho_0$ and the volume fraction of comb-like copolymers, $\phi = n_1\mathrm{N/V}\rho_0$. The repulsive interaction between A and B components is characterized by the product of $\chi N$ with the Flory-Huggins parameter $\chi$. The spatial distributions of volume fractions of A- and B-components, $\phi_{\mathrm{A}}(\mathbf{r})$ and $\phi_{\mathrm{B}}(\mathbf{r})$ are chosen to characterize the ordered phases self-assembled in this blend. Note that $\phi_{\mathrm{A}}(\mathbf{r})$ consists of the volume fractions of A-blocks (denoted as $\phi_{\mathrm{A}}^{\mathrm{C}}(\mathbf{r})$ ) and A-homopolymers (denoted as $\phi_{\mathrm{H}}(\mathbf{r})$ ), ie. $\phi_{\mathrm{A}}(\mathbf{r}) = \phi_{\mathrm{A}}^{\mathrm{C}}(\mathbf{r}) + \phi_{\mathrm{H}}(\mathbf{r})$. We define a parameter of $\tau_i$ to describe the location of the $i$-th graft in the comb-like copolymer with $m$ grafts, given by

$$\tau_i = \tau_1 + \frac{(i-1)(1-2\tau_1)}{m-1} \quad 1 \le i \le m, \tag{S1}$$

The block length between neighboring junctions is denoted by $\Delta\tau = (1-2\tau_1)/(m-1)$ (in units of $N_{\mathrm{A}}$).

Under the approximations of the mean-field treatment and Gaussian-chain model, the free energy in the canonical ensemble can be expressed as

$$\begin{aligned} \frac{NF}{\rho_0 V k_{\mathrm{B}} T} &= -\phi \ln \frac{Q_{\mathrm{C}}}{\phi} - \frac{1-\phi}{\gamma} \ln \frac{Q_{\mathrm{H}}}{1-\phi} \\ &+ \frac{1}{V}\int \mathrm{d}\mathbf{r}\{\chi N \phi_{\mathrm{A}}(\mathbf{r})\phi_{\mathrm{B}}(\mathbf{r}) - \omega_{\mathrm{A}}(\mathbf{r})\phi_{\mathrm{A}}(\mathbf{r}) - \omega_{\mathrm{B}}(\mathbf{r})\phi_{\mathrm{B}}(\mathbf{r}) \\ &- \eta(\mathbf{r})[1-\phi_{\mathrm{A}}(\mathbf{r}) - \phi_{\mathrm{B}}(\mathbf{r})]\} \end{aligned} \tag{S2}$$

where $\omega_{\mathrm{K}}(\mathbf{r})$ is the mean-field conjugating to the volume fraction $\phi_{\mathrm{K}}(\mathbf{r})$ ( K= A or B). The spatial function $\eta(\mathbf{r})$ is a Lagrange multiplier used to enforce the incompressibility condition, $\phi_{\mathrm{A}}(\mathbf{r})$ + $\phi_{\mathrm{B}}(\mathbf{r}) = 1$ . The two quantities $Q_{\mathrm{C}}$ and $Q_{\mathrm{H}}$ are the partition functions of single copolymer chain and single homopolymer chain interacting with the mean fields of $\dot{w}_{\mathrm{A}}(\mathbf{r})$ and $w_{\mathrm{B}}(\mathbf{r})$ respectively. $Q_H$,$Q_C$ is determined by

$$Q_{\mathrm{H}} = \frac{1}{V}\int \mathrm{d}\mathrm{r}\, q_{\mathrm{H}}(\mathbf{r}, s = 1) \tag{S3}$$

$$Q_{\mathrm{C}} = \frac{1}{V}\int \mathrm{d}\mathrm{r}\, q_{\mathrm{A}}(\mathbf{r}, s = 1) \tag{S4}$$

Here, $q_{\mathrm{H}}(\mathbf{r},s)$ is the propagator function for the homopolymer starting from one of its two equivalent free ends while the $q_{\mathrm{A}}(\mathbf{r},s)$ is that for the backbone of the comb-like copolymer. The propagator function $q_{\mathrm{H}}(\mathbf{r},s)$ satisfies the following modified diffusion equation:

$$\frac{\partial q_{\mathrm{H}}(\mathbf{r},s)}{\partial s} = \nabla^2 q_{\mathrm{H}}(\mathbf{r},s) - \omega_{\mathrm{A}}(\mathbf{r}) q_{\mathrm{H}}(\mathbf{r},s) \tag{S5}$$

In the above equation, the radius of gyration of an unperturbed linear polymer with $N$ segments, $R_g = (N/6)^{1/2}b$, is chosen as the unit of spatial length. Standard initial conditions of the propagator functions for the homopolymer are used, e.g $q_{\mathrm{H}}(\mathbf{r},0) = 1$.

For the comb-like copolymer, the contour length increases continuously from 0 (corresponding to one end of the backbone chain) to 1 (correspondingto the other end of the backbone chain). The backbone propagator is divided into $m+1$ segments

$$q_{\mathrm{A}}(\mathbf{r},s) = q_{\mathrm{A}}^{(j)}(\mathbf{r},s) \tag{S6}$$

$$\text{for } \tau_j \le s < \tau_{j+1}; j = 0,\, 1, ..., m;\ \tau_0 \equiv 0,\ \tau_{m+1} \equiv 1 \tag{S7}$$

where each segment satisfies the modified diffusion equation

$$\frac{N}{N_{\mathrm{A}}}\frac{\partial q_{\mathrm{A}}^{(j)}(\mathbf{r},s)}{\partial s}=\nabla^2 q_{\mathrm{A}}^{(j)}(\mathbf{r},s)-\omega_{\mathrm{A}}(\mathbf{r})q_{\mathrm{A}}^{(j)}(\mathbf{r},s) \tag{S8}$$

and is subject to the following initial conditions

$$q_{\mathrm{A}}^{(j)}(\mathbf{r},\tau_j)=q_{\mathrm{A}}^{(j-1)}(\mathbf{r},\tau_j)q_{\mathrm{B}}(\mathbf{r},1);j=1,2,...,m;q_{\mathrm{A}}^{(0)}(\mathbf{r},0)=1 \tag{S9}$$

Here, $q_{\mathrm{B}}(\mathbf{r},s)$ is the propagator of B graft block, descrubed by the following modified diffusion equation

$$\frac{N}{N_{\mathrm{B}}}\frac{\partial q_{\mathrm{B}}(\mathbf{r},s)}{\partial s}=\nabla^2 q_{\mathrm{B}}(\mathbf{r},s)-\omega_{\mathrm{B}}(\mathbf{r})q_{\mathrm{B}}(\mathbf{r},s) \tag{S10}$$

and is subject to the initial condition $q_{\mathrm{B}}(\mathbf{r},0)=1$ for the free end of the graft at $s=0$ .We also define a back-propagator of the $j$-th B chain, $q_{\mathrm{B}j}^{+}(\mathbf{r},s)$ . It satisfies eq S10 and starts on the end of the B chain tethered to the backbone.It is therefore subject to the initial condition

$$q_{\mathrm{B}j}^{+}(\mathbf{r},0)=\frac{q_{\mathrm{A}}^{(j)}(\mathbf{r},\tau_j)q_{\mathrm{A}}^{(j)}(\mathbf{r},1-\tau_j)}{q_{\mathrm{B}}^{2}(\mathbf{r},1)} \tag{S11}$$

In terms of these propagators, the monomer densities $\varphi_{\mathrm{A}}(\mathbf{r})$ and $\varphi_{\mathrm{B}}(\mathbf{r})$ become

$$\begin{aligned}\varphi_{\mathrm{A}}(\mathbf{r})&=\sum_{i=1}^{m+1}\varphi_{\mathrm{A}}^{i}(\mathbf{r})+\varphi_{\mathrm{H}}(\mathbf{r})\\&=\frac{\phi f_{\mathrm{A}}}{Q_{\mathrm{C}}}\sum_{i=1}^{m+1}\int_{\tau_{i-1}}^{\tau_i}\mathrm{d}s\,q_{\mathrm{A}}(\mathbf{r},s)q_{\mathrm{A}}(\mathbf{r},1-s)+\frac{1-\phi}{\gamma Q_{\mathrm{H}}}\int_0^{\gamma}\mathrm{d}s q_{\mathrm{H}}(\mathbf{r},\,s)q_{\mathrm{H}}(\mathbf{r},\,\gamma-s)\end{aligned} \tag{S12}$$

$$\varphi_{\mathrm{B}}(\mathbf{r})=\frac{\phi f_{\mathrm{B}}}{mQ_{\mathrm{C}}}\sum_{j=1}^{m}\int_0^1\mathrm{d}s\,q_{\mathrm{B}}(\mathbf{r},s)q_{\mathrm{B}j}^{+}(\mathbf{r},1-s) \tag{S13}$$

where, $\varphi_{\Lambda}^{i}(\mathbf{r})$ is the density coming from blocks between $\tau_{i-1}$ and $\tau_i$. Finally,the minimization of the free energy with respect to $\phi_{\mathrm{k}}(\mathbf{r})$ and $w_{\mathrm{K}}(\mathbf{r})$ leads to the following mean-field equations:

$$\omega_{\mathrm{A}}(\mathbf{r}) = \chi N \varphi_{\mathrm{B}}(\mathbf{r}) + \eta(\mathbf{r})$$

$$\omega_{\mathrm{B}}(\mathbf{r}) = \chi N \varphi_{\mathrm{A}}(\mathbf{r}) + \eta(\mathbf{r})$$

$$\varphi_{\mathrm{A}}(\mathbf{r}) + \varphi_{\mathrm{B}}(\mathbf{r}) = 1$$

The SCFT equations are solved numerically using a standard iteration scheme, and the second-order pseudospectral method is empolied to solve the modified diffusion equations. The contour step size is set as $\Delta s = 0.01$.
